# Associations of “weekend warrior” and other physical activity patterns with sarcopenia among older adults in China: a cross-sectional study

**DOI:** 10.1186/s12889-026-27243-1

**Published:** 2026-04-09

**Authors:** Shuomin Wang, Pui Hing Chau, Mengting Dai, Kehan Liu, Wenru Wang, Minhui Liu, Chia-Chin Lin

**Affiliations:** 1 School of Nursing, Li Ka Shing Faculty of Medicine, The University of Hong Kong, 5/F, Academic Building, 21 Sassoon Road, Pok Fu Lam, Hong Kong SAR China; 2 School of Nursing, Ningxia Medical University, Yinchuan, China; 3 Xiangya School of Nursing, Central South University, Changsha, China; 4 Alice Lee Centre for Nursing Studies, Yong Loo Lin School of Medicine, National University of Singapore, Singapore, Singapore

**Keywords:** Sarcopenia, Physical activity patterns, Weekend warriors, Regularly active

## Abstract

**Background:**

Moderate-to-vigorous physical activity (MVPA) is effective in preventing sarcopenia. Current physical activity guidelines recommend ≥ 150 min/week of moderate-to-vigorous physical activity (MVPA) for health benefits. Based on volume and frequency, distinct MVPA patterns exist beyond the regularly active pattern. The “weekend warrior” pattern, which concentrates 150 min into one or two sessions, may represent an alternative regimen for sarcopenia prevention. This study examined associations between MVPA patterns and risk of sarcopenia in older Chinese adults.

**Methods:**

This cross-sectional study was based on 3,579 participants aged ≥ 60 years in the China Health and Retirement Longitudinal Study (CHARLS). MVPA patterns were self-reported through questionnaires and categorized into four groups: inactive (no MVPA), insufficiently active (IA) (< 150 min of MVPA /week), weekend warrior (WW) (≥ 150 min of MVPA with ≤ 2 sessions/week), and regularly active (RA) (≥ 150 min of MVPA with > 2 sessions/week). Sarcopenia was operationalized based on the 2019 Asian Working Group for Sarcopenia (AWGS) criteria, incorporating the three components of muscle strength, physical performance, and appendicular skeletal muscle mass (ASM). Multivariable logistic regression models were employed to examine the associations of MVPA patterns with odds of sarcopenia, with subgroup analyses evaluating robustness across sociodemographic, lifestyle, and health-related characteristics.

**Results:**

Participants were classified as inactive (*n* = 1492, 41.7%), IA (*n* = 175, 4.9%), WW (*n* = 131, 3.7%) and RA (*n* = 1781, 49.8%). Compared to inactive individuals, those engaging in the WW pattern (OR 0.62, 95% CI: 0.42–0.94), and RA pattern (OR 0.63, 95% CI: 0.53–0.74) showed significantly lower odds of sarcopenia in the fully adjusted model. However, the IA group did not exhibit significant association across any of the models. Subgroup analyses revealed that there were no significant moderators (all interaction *P* > 0.05).

**Conclusions:**

The WW pattern associates with lower odds of sarcopenia than the inactive group, offering a potential alternative for older adults who are unable to maintain regular weekly physical activity. These findings support incorporating the WW pattern into flexible physical activity recommendations for sarcopenia prevention in older adults.

**Supplementary Information:**

The online version contains supplementary material available at 10.1186/s12889-026-27243-1.

## Introduction

Sarcopenia, characterized by the age-related loss of skeletal muscle mass plus loss of muscle strength and/or reduced physical performance, has emerged as a major public health concern among older adults [1]. In China, the prevalence of sarcopenia is approximately 20.7% of individuals aged 60 years and older, with even higher rates observed in the oldest age groups [2]. This geriatric syndrome is associated with adverse health outcomes, including physical disability, frailty, increased risk of falls, and mortality [3]. Therefore, it is of great importance to identify modifiable factors to prevent or mitigate sarcopenia.

Physical activity (PA), especially moderate-to-vigorous physical activity (MVPA), is a well-established important factor against sarcopenia [4, 5]. Sufficient PA can enhance muscle protein synthesis, improves neuromuscular function, and reduces age-related muscle atrophy. Over the past decade, PA guidelines have shifted toward a more flexible framework that emphasizes the total weekly accumulation of MVPA. The World Health Organization (WHO) recommends that older adults engage in at least 150 min of moderate-intensity PA (MPA), or 75 min of vigorous PA (VPA), or an equivalent combination each week [6]. However, achieving consistent daily PA remains challenging for many individuals due to time constraints or sedentary lifestyles. This has led to the rise of alternative PA patterns, such as the “weekend warrior (WW)” model—where individuals condense their PA into one or two intensive sessions within a week [7]. From a behavioral perspective, this approach may offer a more feasible and practical strategy for meeting PA targets, accommodating variability in time availability and motivation while still conferring substantial health benefits.

Emerging evidence suggests that the WW pattern can yield health benefits comparable to those of regular PA, including reduced risks of cardiovascular disease [8], neurodegenerative diseases [9], and all-cause mortality [10]. These findings suggest that PA volume, rather than frequency, may be the predominant determinant of health outcomes. While regularly distributed MVPA is known to mitigate risk of sarcopenia through sustained anabolic stimulation and metabolic regulation [11, 12], it remains unclear whether short-duration, high-volume sessions (such as the WW pattern) provide similar protective benefits. Given the growing prevalence of sarcopenia and the practical appeal of time-efficient activity models, elucidating their relationship is critical for public health recommendations.

To address this gap, this cross-sectional study aimed to investigate the association between MVPA patterns and prevalence of sarcopenia. We hypothesized that concentrated PA sessions (i.e., the WW pattern) would also provide reduced odds of sarcopenia, comparing to the inactive group. Our findings might provide new insights into how variations in MVPA distribution influence muscle health and to inform the development of tailored PA recommendations for sarcopenia prevention.

## Methods

### Study population

The China Health and Retirement Longitudinal Study (CHARLS) is a nationally longitudinal cohort study aimed at examining the health and socioeconomic conditions associated with aging. The CHARLS sample is representative of adults aged 45 and over who live in households. Utilizing a multistage, stratified, probability-proportional-to-size (PPS) sampling design, this survey covers 28 provinces, 150 counties/districts, and 450 villages/urban committees. The baseline survey was conducted in 2011, enrolling 17,708 participants from 10,257 households. Follow-up assessments were conducted in 2013, 2015, 2018, and 2020. Data were collected through face-to-face interviews using computer-assisted personal interviewing (CAPI) technology, capturing detailed information on sociodemographic characteristics, economic status, and health-related indicators. The project was approved by the Biomedical Ethics Committee of Peking University (IRB00001052-11015) and adhered to the Declaration of Helsinki [13].

**Fig. 1:**
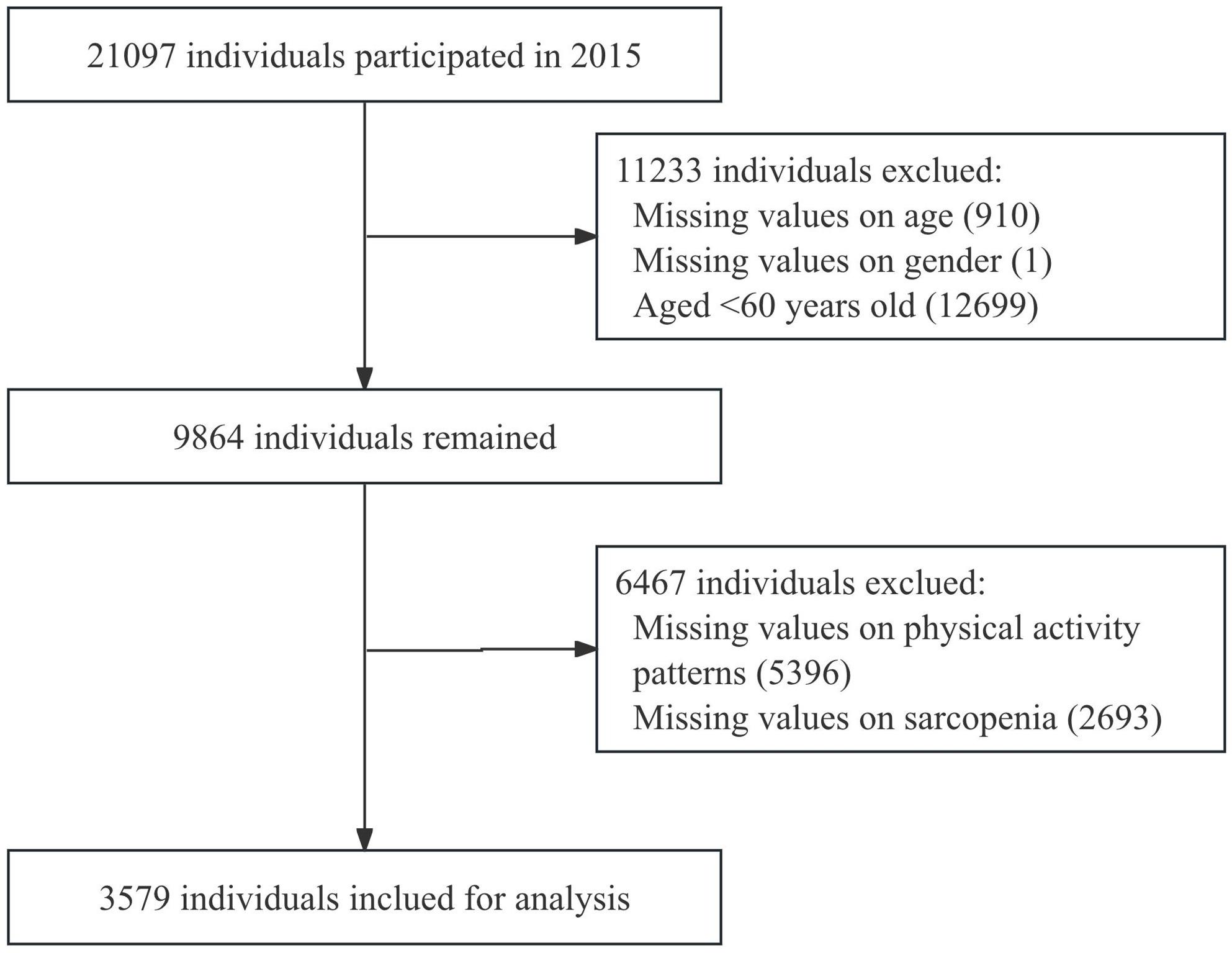
Flowchart of the study

Because measurements of sarcopenia components were conducted only during the first three waves of CHARLS, and a larger proportion of respondents provided information on PA in Wave 3, the present analysis was based on data from Wave 3 (2015). The initial sample in 2015 included 21,097 individuals, and we restricted the analysis to participants aged 60 years and above. Participants were excluded from the analysis if they had: (1) missing information on age or gender; (2) incomplete assessment of PA; and (3) missing data on sarcopenia components. Compared to excluded participants, the final analytic sample was generally younger and exhibited better physical health status. The participant selection flowchart is presented in Fig. 1.

### Independent variable

In CHARLS, physical activity (PA) was assessed using a modified version of the International Physical Activity Questionnaire - Short Form (IPAQ-SF) [14], including: vigorous-intensity activities (VPA), moderate-intensity activities (MPA), and light-intensity activities (LPA). VPA refers to activities that cause individuals to breathe much harder than normal, such as heavy lifting, digging, plowing, aerobics, fast bicycling, or cycling with a heavy load. MPA refers to activities that lead to somewhat harder breathing than usual, including carrying light loads, bicycling at a regular pace, or mopping the floor. More details are provided in the Appendix.

For each activity type, participants who responded affirmatively were further asked to report: (1) the number of days per week (1–7 days), and (2) the average duration per day, categorized into four intervals: 10–30 min, 30 min–2 h, 2–4 h, and ≥ 4 h. In accordance with established methodology [15, 16], these categorical responses were converted to continuous values using the midpoint of each interval: 20, 75, 180, and 240 min, respectively. Subsequently, MVPA was calculated by summing the total minutes of moderate activity and twice the minutes of vigorous activity. Based on weekly MVPA levels and frequency, participants were categorized into four MVPA patterns: inactive (no MVPA), insufficiently active (IA) (< 150 MVPA minutes/week), weekend warrior (≥ 150 MVPA minutes/week with ≤ 2 sessions/week), and regularly active (≥ 150 MVPA minutes/week with > 2 sessions/week) [7, 8].

### Dependent variable

Sarcopenia was diagnosed based on the 2019 criteria established by the Asian Working Group for Sarcopenia (AWGS) [1], incorporating three core components: muscle strength, physical performance, and appendicular skeletal muscle mass (ASM). In community preventive services settings, possible sarcopenia was defined as low muscle strength, with or without low physical performance, while a confirmed sarcopenia diagnosis required low muscle mass combined with either low muscle strength or poor physical performance. For practical application in community-based health promotion, participants were categorized into two groups: those with sarcopenia (including possible or confirmed cases) and those without.

Muscle strength was assessed using a Yuejian™ WL-1000 dynamometer. Participants performed the grip strength test in a standing position, holding the dynamometer at a right angle, and squeezing the handle with maximal effort for a few seconds. Participants performed two tests for each hand, and the maximal grip strength with the dominant hand was used for analysis. Gender-specific cut-off values were applied to define low muscle strength: <28 kg for males and < 18 kg for females [1].

Physical performance was evaluated through five-time chair stand tests and gait speed. Participants were instructed to complete five consecutive rises from a 47-cm-high armless chair with arms crossed over the chest; the total time from the initial standing to the final seated position was recorded. In addition, participants completed two walks along a 2.5-meter course at their usual pace, and the average gait speed (in m/s) was calculated. Low physical performance was defined as a chair stand time ≥ 12 s or a gait speed < 1.0 m/s [1].

Appendicular skeletal muscle mass (ASM) was estimated using a validated anthropometric equation specifically developed for the Chinese population [17]: ASM (kg) = 0.193 × weight (kg) + 0.107 × height (cm) − 4.157 × gender (1 = male, 2 = female) − 0.037 × age − 2.631, where gender was coded as 1 for males and 2 for females. Body weight and height were measured using standardized equipment: an Omron™ HN-286 digital scale and a Seca™ 213 portable stadiometer, respectively. Low muscle mass was defined as height-adjusted ASM (ASM/height^2^, kg/m^2^) falling below gender-specific thresholds corresponding to the lowest quintile of the study population [1]: <6.90 kg/m^2^ for males and < 5.08 kg/m^2^ for females for our analysis.

### Covariates

Referring to previous published studies, potential covariates covered sociodemographic characteristics, lifestyle variables, and health-related variables [18–21]. Sociodemographic characteristics included age (60–69 vs. ≥70 years), gender, marital status (married vs. unmarried), educational attainment (elementary school or below, middle school, and high or above), place of residence (rural vs. urban), and current employment status (yes/no). Current smoking and alcohol use were dichotomized (yes/no). Night sleep duration was evaluated and classified into ≤6 h, 7–8 h, ≥9 h [19]. Body mass index (BMI) was categorized into four groups according to the WHO recommendation for Asian population [22]: underweight (< 18.5 kg/m^2^), normal (≥ 18.5 and < 23 kg/m^2^), overweight (≥ 23 and < 25 kg/m^2^), and obesity (≥ 25 kg/m^2^). The number of self-reported comorbidities was also assessed, including conditions such as hypertension, hyperglycemia, dyslipidemia, cancer, chronic lung diseases, heart disease, stroke, emotional or mental disorders, arthritis, hepatic disease, kidney disease, digestive system diseases, and asthma. Based on the total count, comorbidities were categorized as 0, 1, or ≥ 2.

### Statistical analysis

Continuous variables were presented as mean ± standard deviation, while categorical variables were presented as frequencies (percentages). Between-group comparisons across four MVPA patterns were conducted using one-way analysis of variance (ANOVA) for continuous variables and chi-square tests for categorical variables. Logistic regression analyses were employed to examine the association between MVPA patterns and sarcopenia. Odds ratios (ORs) and 95% confidence intervals (CIs) were reported. Three models were constructed using a sequential adjustment strategy to evaluate the robustness of the association between MVPA patterns and sarcopenia while progressively accounting for potential confounders. Model 1 was an unadjusted (crude) model. It serves as a baseline reference for subsequent models. Model 2 was adjusted for sociodemographic variables, including age, gender, marital status, educational attainment, place of residence, and employment status. Model 3 was further adjusted for lifestyle and health-related variables, including smoking status, drinking status, night sleep duration, BMI, and number of comorbidities. This hierarchical approach allows us to assess how the inclusion of different covariates influences the observed associations and to identify whether the effect of MVPA patterns on sarcopenia is independent of these factors. Multicollinearity was assessed using the variance inflation factor (VIF) after fitting the fully adjusted model (Model 3). In our study, all variables demonstrated VIF values below 5, with a mean VIF of 1.58, indicating no substantial multicollinearity among the covariates included [23]. In addition, subgroup analyses were performed to explore potential effect modifications based on covariate stratification, with interaction terms tested for statistical significance.

In sensitivity analyses: (1) weighted logistic regression analyses were conducted; (2) missing data on covariates were imputed 20 times using multiple imputation by chained equations (MICE); (3) alternative thresholds of physically active group based on the weekly MVPA (at the level of 100, 125, 175 and 200 min, respectively) [24] were employed to examine whether there would be any change in the association between being a weekend warrior and sarcopenia. This analysis moves beyond the WHO recommendation to investigate whether benefits are sustained at both lower and higher volumes of concentrated activity; and (4) sarcopenia was regarded as a three-categorical variable (no sarcopenia, possible sarcopenia, and confirmed sarcopenia) using multinomial logistic regression models, to examine whether the observed associations with MVPA patterns remained consistent across varying severity levels. All statistical tests were two-sided, and *P* values < 0.05 were considered statistically significant. Analyses were conducted using Stata SE version 16.0 (StataCorp) and R software, version 4.5.1 (R Project for Statistical Computing).

## Results

### Baseline characteristics of participants

Table 1 summarizes the baseline characteristics of participants across different MVPA pattern groups. The analytical sample included 3,579 participants, including 1,492 (41.7%) inactive individuals, 175 (4.9%) IA individuals, 131 (3.7%) in the WW group, and 1,781 (49.8%) in the RA group. Significant between-group differences were observed in age, gender, marital status, educational attainment, residential area, employment status, smoking status, drinking status, sleep duration and comorbidities (all *P* < 0.05). Compared to the inactive counterparts, weekend warriors tended to be younger, predominantly male, more highly educated, currently employed, less likely to smoke or consume alcohol, and had a lower prevalence of chronic conditions.

**Table 1:** Baseline characteristics of participants by MVPA patterns

	Overall (*N* = 3,579)	Inactive (*N* = 1,492)	Insufficiently active (*N* = 175)	Weekend warriors (*N* = 131)	Regularly active (*N* = 1,781)	χ^2^	*P*-values
Age group, *n* (%)							
60-69y	2,335 (65.2)	804 (53.9)	112 (64.0)	102 (77.9)	1,317 (73.9)	153.66	< 0.001
≥70y	1,244 (34.8)	688 (46.1)	63 (36.0)	29 (22.1)	464 (26.1)		
Gender, n (%)						23.19	< 0.001
Male	1,805 (50.4)	728 (48.8)	65 (37.1)	81 (61.8)	931 (52.3)		
Female	1,774 (49.6)	764 (51.2)	110 (62.9)	50 (38.2)	850 (47.7)		
Marital status, n (%)						26.12	< 0.001
Married	2,904 (81.1)	1,161 (77.8)	133 (76.0)	107 (81.7)	1,503 (84.4)		
Unmarried	675 (18.9)	331 (22.2)	42 (24.0)	24 (18.3)	278 (15.6)		
Place of residence, n (%)						54.51	< 0.001
Urban	1,269 (35.5)	576 (38.6)	98 (56.0)	42 (32.1)	553 (31.0)		
Rural	2,310 (64.5)	916 (61.4)	77 (44.0)	89 (67.9)	1,228 (69.0)		
Education attainment, n (%) ^#^						15.44	0.017
Elementary school or below	2,890 (80.8)	1,219 (81.7)	126 (72.0)	106 (80.9)	1,439 (80.8)		
Middle school	462 (12.9)	174 (11.7)	38 (21.7)	15 (11.5)	235 (13.2)		
High school or above	226 (6.3)	99 (6.6)	11 (6.3)	10 (7.6)	106 (6.0)		
Employment status, n (%) ^#^						459.51	< 0.001
No	1,585 (44.3)	945 (64.5)	103 (59.2)	51 (39.5)	486 (27.5)		
Yes	1,947 (54.4)	519 (35.5)	71 (40.8)	78 (60.5)	1,279 (72.5)		
Current smoking status, n (%) ^#^						18.23	< 0.001
No	2,569 (71.8)	1,102 (73.9)	141 (80.6)	82 (62.6)	1,244 (70.0)		
Yes	1,007 (28.1)	390 (26.1)	34 (19.4)	49 (37.4)	534 (30.0)		
Current drinking status, n (%) ^#^						48.80	< 0.001
No	2,413 (67.4)	1,091 (73.2)	128 (73.1)	90 (68.7)	1,104 (62.0)		
Yes	1,164 (32.5)	400 (26.8)	47 (26.9)	41 (31.3)	676 (38.0)		
Night sleep duration, n (%) ^#^						15.26	0.018
≤5 h	1,222 (34.1)	509 (35.3)	69 (40.4)	47 (35.9)	597 (34.0)		
6–8 h	1.925 (53.8)	760 (52.7)	88 (51.5)	74 (56.5)	1,003 (57.2)		
≥9 h	352 (9.8)	174 (12.1)	14 (8.2)	10 (7.6)	154 (8.8)		
BMI, n (%) ^#^						15.40	0.081
Underweight	282 (7.9)	130 (8.9)	13 (7.5)	6 (4.6)	133 (7.5)		
Normal	1,439 (40.2)	559 (38.2)	61 (35.3)	58 (44.3)	761 (43.0)		
Overweight	724 (20.2)	295 (20.2)	39 (22.5)	29 (22.1)	361 (20.4)		
Obesity	1,090 (30.5)	479 (32.7)	60 (34.7)	38 (29.0)	513 (29.0)		
Comorbidity, n (%)						14.85	0.021
0	1,146 (32.0)	462 (31.0)	39 (22.3)	46 (35.1)	599 (33.6)		
1	1,030 (28.8)	437 (29.3)	51 (29.1)	43 (32.8)	499 (28.0)		
≥2	1,403 (39.2)	593 (39.7)	85 (48.6)	42 (32.1)	683 (38.3)		

^#^ 1 missing on education attainment, 3 missing on smoking status, 47 missing on employment status, 2 missing on drinking status, 80 missing on sleep duration, and 40 missing on BMI. *MVPA* moderate-to-vigorous physical activity, *BMI* body mass index

### Association between MVPA patterns and sarcopenia

The results of logistic regression analyses were presented in Table 2. Using the inactive group as the reference category, both regularly active participants and weekend warriors demonstrated a significantly lower odds of sarcopenia in the unadjusted models, with ORs of 0.46 (95% CI: 0.40–0.53) and 0.44 (95% CI: 0.30–0.65), respectively. These associations remained statistically significant, albeit attenuated, in subsequent models after adjusting for sociodemographic factors (OR 0.62, 95% CI: 0.53–0.72 for the RA pattern; OR 0.60, 95% CI: 0.40–0.89 for the WW pattern) and further adjustment for health-related covariates (OR 0.63, 95% CI: 0.53–0.74 for the RA pattern; OR 0.62, 95% CI: 0.42–0.94 for the WW pattern). However, the IA group did not exhibit significantly lower odds of sarcopenia compared to the inactive group across any of the models.

**Table 2:** Associations between MVPA patterns and sarcopenia among older adults

PA patterns	Model 1 OR (95% CI)	*P*-values	Model 2 OR (95% CI)	*P*-values	Model 3 OR (95% CI)	*P*-values
Inactive	Reference		Reference		Reference	
Insufficiently active	0.74 (0.54, 1.01)	0.061	0.87 (0.63, 1.22)	0.430	0.82 (0.58, 1.16)	0.272
Weekend warriors	0.44 (0.30, 0.65)	< 0.001	0.60 (0.40, 0.89)	0.012	0.62 (0.42, 0.94)	0.023
Regularly active	0.46 (0.40, 0.53)	< 0.001	0.62 (0.53, 0.72)	< 0.001	0.63 (0.53, 0.74)	< 0.001

*MVPA* moderate-to-vigorous physical activity, *OR* odd ratio, *CI* confidence interval

Model 1: No covariates were adjusted

Model 2: Adjusted for age, gender, marital status, educational attainment, place of residence, and employment status

Model 3: Adjusted for Model 2 + smoking status, drinking status, night sleep duration, body mass index (BMI), and number of comorbidities

### Subgroup analysis

**Fig. 2:**
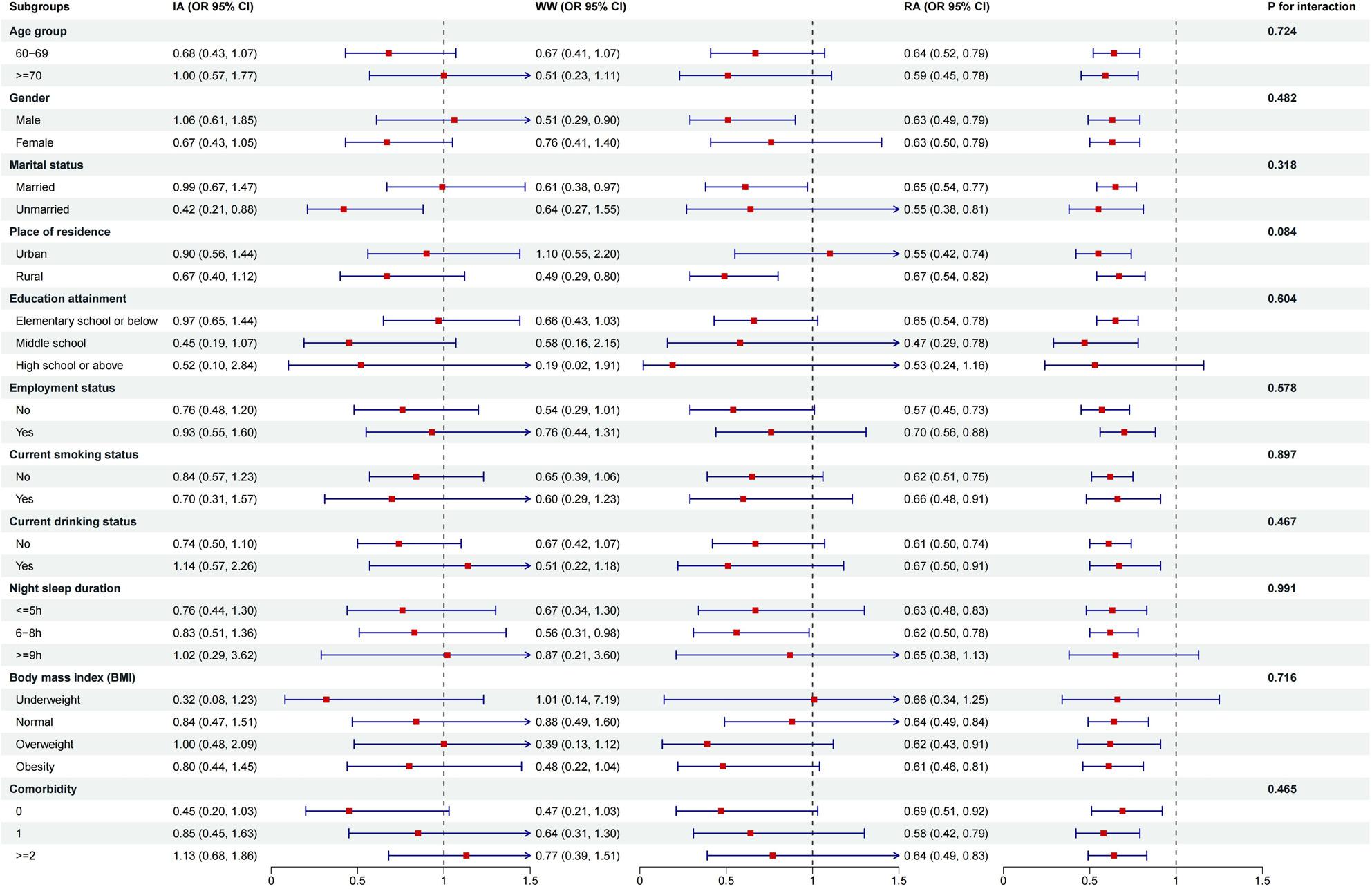
Subgroup analyses for the association between MVPA pattern and sarcopenia among older adults. MVPA moderate-to-vigorous physical activity, OR odd ratio, CI confidence interval, IA insufficiently active, WW weekend warriors, RA regularly active. The reference group was inactive participants (no MVPA). All analyses were adjusted for age, gender, marital status, educational attainment, place of residence, smoking status, drinking status, body mass index, and number of comorbidities. Interaction term was tested by Wald test

As illustrated in Fig. 2, the WW pattern was significantly associated with a reduced odds of sarcopenia compared to the inactive group, among male participants, married individuals, rural residents, and those reporting 6–8 h of nocturnal sleep. The RA pattern showed significantly lower odds of sarcopenia across all subgroups, except for individuals with an educational attainment of high school or above, those with ≥ 9 h of sleep per night, and underweight participants. However, no statistically significant interactions were observed between MVPA patterns and the subgroups defined by age group, gender, marital status, place of residence, education attainment, employment status, smoking status, drinking status, sleep duration, BMI, and comorbidity on odds of sarcopenia (all *P* for interaction > 0.05).

### Sensitivity analyses

The findings of sensitivity analyses were largely consistent with the primary results when sampling weights were incorporated (Supplementary Table 1), and missing covariate data were imputed (Supplementary Table 2). When defining an active MVPA pattern as achieving ≥ 100 min/week of MVPA, both the WW pattern (OR 0.62, 95% CI 0.42–0.94) and RA pattern (OR 0.63, 95% CI 0.54–0.74) exhibited statistically significant reductions in odds of sarcopenia compared to the inactive group (Supplementary Table 3). This association persisted at a higher MVPA threshold of 125 min/week, with both PA patterns demonstrating a similarly lower odds (WW: OR 0.63, 95% CI 0.42–0.94; RA: OR 0.64, 95% CI 0.54–0.75). However, at more stringent MVPA thresholds (175 and 200 min/week), the WW pattern no longer conferred a significant reduction in odds of sarcopenia (OR 0.75, 95% CI 0.47–1.20 and OR 0.74, 95% CI 0.46–1.18, respectively). Additionally, while the WW pattern consistently showed a reduced odds of possible sarcopenia (OR 0.58, 95% CI 0.37–0.89) comparing to the inactive group, this association did not reach statistical significance for confirmed sarcopenia (OR 0.99, 95% CI 0.44–2.22) (Supplementary Table 4).

## Discussion

Our study investigated the association between weekly MVPA patterns and prevalence of sarcopenia in Chinese older adults. We found that both the WW and RA PA patterns, but not the IA pattern, were associated with lower odds of sarcopenia. The results of sensitivity analyses confirmed the robustness of our findings. Our study highlights that the choice of concentrated MVPA over 1–2 sessions throughout the week may reduce the possibility of developing sarcopenia, thereby expanding the range of feasible PA options for older adults.

Existing research has established the important role of regular PA, particularly MVPA, against sarcopenia [25–27]. Based on the minimum recommendation of WHO guidelines for integrated MVPA volume [6], several recent studies have extended this framework by investigating how distinct patterns of MVPA accumulation influence sarcopenia risk. One cross-sectional study reported that WWs had 84% lower odds of prevalent sarcopenia compared to inactive individuals in middle-aged and older adults [21]. Another prospective cohort study demonstrated that the WW pattern was associated with a 21% to 26% reduced risk of developing incident sarcopenia over a median of 7.8 years [20]. Aligning with this body of evidence, our study demonstrated that both the WW and RA patterns confer reduced odds of sarcopenia compared to physical inactivity. Collectively, these findings suggest that the total weekly volume of activity may be more critical than its temporal distribution for sarcopenia prevention.

An unexpected finding of our study was that the IA group (ranging from 1 to 149 min/week) did not demonstrate significantly lower odds of sarcopenia compared to the inactive group. This finding contrasts with the widely accepted premise that PA confers health benefits, even in brief bouts [28]. A potential explanation is related to the heterogeneity of the IA group, as individuals at the lower end of this spectrum may derive minimal muscle health benefits. However, this null finding can be supported by another longitudinal study, which found that taking 10–150 min/week on VPA, but not on MVP, was linked to lower sarcopenia risk [29]. Taken together, the relationship between low-volume MVPA and sarcopenia may need further investigation.

Despite the non-significant interaction, subgroup analysis indicated that the benefits of the WW pattern were statistically significant only in certain subgroups. For example, male participants appeared more likely to benefit from the WW pattern. This finding contrasts with a previous study reporting that the inverse association between the WW pattern and sarcopenia was more pronounced in women [20]. This discrepancy may be attributable to differences in study design or sample characteristics. For the other subgroups, the benefits of the WW pattern were statistically significant in married individuals, rural residents, and those with moderate sleep duration (6–8 h), but not in their respective counterparts. Married individuals have a lower risk of sarcopenia, potentially due to greater social support and healthier lifestyle behaviors [30]. Similarly, rural residents showed a lower odds of sarcopenia, since they could engage in more farming activities which are regarded as a form of horticultural therapy and improve subjective well-being and physical function [31]. Additionally, both short and long sleep duration are associated with increased sarcopenia risk [19]. It suggests that moderate sleep duration may represent an optimal physiological state for muscle maintenance and recovery. Thus, it is plausible that individuals in these subgroups, who already exhibit a lower baseline risk of sarcopenia, may be better positioned to derive additional benefits from the WW pattern. However, these findings warrant cautious interpretation. The absence of significant interactions indicates these variables might not be true effect modifiers, and the subgroup-specific significance could also be influenced by limited statistical, particularly given the relatively small sample size of the WW group.

There is ongoing debate regarding the optimal volume of PA for different populations and health outcomes [32, 33], and the dose-response relationship between PA and sarcopenia risk remains inconclusive [34–36]. Hence, our study applied different thresholds of being physically active, which deviate from the WHO recommendation of at least 150 min of MVPA per week. The significant association between the WW pattern and lower sarcopenia prevalence was observed at the thresholds of 100 and 125 min per week, but not at the higher thresholds of 175 and 200 min (Supplementary Table 3). However, the insignificant association at 175 min/week threshold contrasts with previous research on type 2 diabetes [24], which reported risk reductions among weekend warriors even at 175 min/week. An explanation concerns feasibility and statistical power: achieving the substantially higher volumes of 175–200 min of MVPA within only one or two weekly sessions is somewhat impractical, resulting in fewer participants classified as WW at these thresholds and thus reducing the ability to detect a statistically significant association. On the other hand, this threshold-dependent effect was partially supported by emerging evidence of a nonlinear dose-response relationship between PA and sarcopenia risk [35, 36], implying that there may be an optimal range of concentrated activity volume for maximizing benefits. Future studies with larger samples of high-volume WW participants are needed to further explore these potential nonlinear relationships. In addition, we found that the WW pattern was associated with possible sarcopenia, but not with confirmed sarcopenia (Supplementary Table 4). One explanation is that possible sarcopenia represents an earlier, less severe stage of muscle deterioration where lifestyle interventions like PA may exert more pronounced effects.

PA exerts protective effects against sarcopenia through distinct yet complementary physiological mechanisms that vary by exercise modality [37]. For example, aerobic exercise enhances mitochondrial ATP production in skeletal muscle, downregulates catabolic gene expression while increasing muscle protein synthesis [38]; resistance exercise stimulates muscle hypertrophy and enhances strength by altering the balance between muscle protein synthesis and degradation [39]. Although our study did not differentiate between specific exercise types, the reduced odds of sarcopenia observed in the WW pattern may be attributed to its ability to deliver adequate mechanical and metabolic stimuli during concentrated sessions. While exercise-based intervention programs typically recommend frequencies of ≥ 3 sessions per week, such regimens may present adherence challenges for some older adults [40]. Our results suggest that the WW pattern might offer a viable alternative, potentially improving compliance while achieving meaningful health benefits. However, the implementation of such concentrated training approaches requires careful risk-benefit consideration. Current evidence indicates potential increased susceptibility to musculoskeletal injuries (particularly in shoulders, elbows, and ankles) with the WW pattern [41, 42]. It highlights the need for appropriate warm-up protocols, proper technique instruction, and gradual intensity progression. Therefore, future research should explore the optimal composition of the WW activity programs, including types, intensity progression, and injury prevention strategies, to maximize both efficacy and safety in older adults.

Limitations should be acknowledged in this study. First, the exclusion of more than half of the initial participants due to missing baseline data resulted in a final sample that was younger and healthier. This may have introduced selection bias. Second, the sample size was imbalanced across MVPA pattern groups, with the RA group being substantially larger than the WW and IA groups. Although the higher proportion of the RA pattern among older adults has been similarly reported in other populations [43], the imbalance representativeness may threaten external validity and generalizability of our findings. Third, due to the cross-sectional study design, causal relationships between different MVPA patterns and sarcopenia cannot be deduced. Forth, the self-reported measurement of PA may be not as accurate as the measurement of PA via accelerometry since self-reports may overestimate duration and intensity of PA, as reported in previous studies [24, 44]. Fifth, we employed an anthropometric equation rather than direct measurement techniques such as bioelectrical impedance analysis or dual X-ray absorptiometry. This methodological choice may have resulted in discrepancies between predicted and actual muscle mass values. Finally, although various potential confounding factors have been adjusted, other potential factors such as dietary status and nutritional supplements [45, 46] which could influence the relationship between PA and sarcopenia were not accounted for in our analyses.

## Conclusion

In conclusion, the WW and RA activity patterns were all associated with similar reductions in the odds of sarcopenia among Chinese older adults, though future research is needed to identify which subpopulations benefit more from the WW pattern. Our findings suggest that achieving the recommended weekly volume of MVPA represents the key determinant for sarcopenia prevention, irrespective of its distribution across the week. The WW pattern may offer a feasible and effective alternative to help preserve muscle health for individuals who are unable to adhere to regularly distributed PA regimens.

## Supplementary Information

Supplementary Material 1.

## Data Availability

The data relevant to this study are available via the CHARLS website (http://charls.pku.edu.cn/en) upon authors’ registration and with the permission of the CHARLS research team.

## Abbreviations

PA: Physical Activity
MVPA: Moderate-to-vigorous Physical Activity
WW: Weekend Warrior
RA: Regularly Active
IA: Insufficiently Active
ASM: Appendicular Skeletal Muscle Mass
BMI: Body Mass Index
