# Supplementary material for: Associations of “weekend warrior” and other physical activity patterns with sarcopenia among older adults in China: a cross-sectional study
Source: BMC Public Health. 2026 Apr 9;26:1619. doi: 10.1186/s12889-026-27243-1 (PMC13191848; doi:10.1186/s12889-026-27243-1)
Supplement: Supplementary file 1 — Supplementary Material 1. [file 12889_2026_27243_MOESM1_ESM.docx]

**Supplementary materials**

**Figure S1. Questionnaire of physical activity used in CHARLS 2015**

**Table S1. Associations between MVPA patterns and sarcopenia among older adults, with adjustment for sampling weights.**

**Table S2. Associations between MVPA patterns and sarcopenia among older adults after imputing missing values on covariates.**

**Table S3. Associations between MVPA patterns and sarcopenia using different activity thresholds of MVPA per week.**

**Table S4. Associations between MVPA patterns and possible/confirmed sarcopenia among older adults.**

**** **Figure S1. Questionnaire of physical activity used in CHARLS 2015**

**Table S1.** **Associations between MVPA patterns and sarcopenia among older adults, with adjustment for sampling weights.**

| **MVPA patterns** | **Model 1**  **OR (95% CI)** | ***P-*values** | **Model 2**  **OR (95% CI)** | ***P-*values** | **Model 3**  **OR (95% CI)** | ***P-*values** |
| --- | --- | --- | --- | --- | --- | --- |
| Inactive | Reference |  | Reference |  | Reference |  |
| Insufficiently active | 0.75 (0.52, 1.08) | 0.118 | 0.86 (0.59, 1.25) | 0.422 | 0.79 (0.53, 1.16) | 0.228 |
| Weekend warriors | 0.45 (0.29, 0.68) | <0.001 | 0.60 (0.38, 0.95) | 0.030 | 0.62 (0.39, 0.98) | 0.040 |
| Regularly active | 0.46 (0.39, 0.55) | <0.001 | 0.60 (0.50, 0.73) | <0.001 | 0.61 (0.50, 0.74) | <0.001 |

MVPA, moderate-to-vigorous physical activity; OR, odd ratio; CI, confidence interval.

Model 1: No covariates were adjusted.

Model 2: Adjusted for age, gender, marital status, educational attainment, place of residence, and employment status.

Model 3: Adjusted for Model 2 + smoking status, drinking status, night sleep duration, body mass index (BMI), and number of comorbidities.

**Table S2. Associations between MVPA patterns and sarcopenia among older adults after imputing missing values on covariates.**

| **MVPA patterns** | **Model 1**  **OR (95% CI)** | ***P-*values** | **Model 2**  **OR (95% CI)** | ***P-*values** | **Model 3**  **OR (95% CI)** | ***P-*values** |
| --- | --- | --- | --- | --- | --- | --- |
| Inactive | Reference |  | Reference |  | Reference |  |
| Insufficiently active | 0.74 (0.54, 1.01) | 0.061 | 0.87 (0.62, 1.22) | 0.415 | 0.84 (0.60, 1.18) | 0.322 |
| Weekend warriors | 0.44 (0.30, 0.64) | <0.001 | 0.58 (0.39, 0.86) | 0.007 | 0.59 (0.40, 0.89) | 0.011 |
| Regularly active | 0.46 (0.40, 0.53) | <0.001 | 0.60 (0.52, 0.71) | <0.001 | 0.60 (0.52, 0.71) | <0.001 |

MVPA, moderate-to-vigorous physical activity; OR, odd ratio; CI, confidence interval.

Model 1: No covariates were adjusted.

Model 2: Adjusted for age, gender, marital status, educational attainment, place of residence, and employment status.

Model 3: Adjusted for Model 2 + smoking status, drinking status, night sleep duration, body mass index (BMI), and number of comorbidities.

**Table S3. Associations between MVPA patterns and sarcopenia using different activity thresholds of MVPA per week.**

|  | **OR (95% CI)** | ***P-*values** |  |
| --- | --- | --- | --- |
| **Active group defined as ≥100 minutes/week of MVPA** | | | |
| Inactive | Reference |  |  |
| Insufficiently active | 0.95 (0.60, 1.51) | 0.832 |  |
| Weekend warriors | 0.62 (0.42, 0.94) | 0.023 |  |
| Regularly active | 0.63 (0.54, 0.74) | <0.001 |  |
| **Active group defined as ≥125 minutes /week of MVPA** | | | |
| Inactive | Reference |  |  |
| Insufficiently active | 0.87 (0.56, 1.36) | 0.543 |  |
| Weekend warriors | 0.63 (0.42, 0.94) | 0.024 |  |
| Regularly active | 0.64 (0.54, 0.75) | <0.001 |  |
| **Active group defined as ≥175 minutes /week of MVPA** | | | |
| Inactive | Reference |  |  |
| Insufficiently active | 0.75 (0.54, 1.03) | 0.071 |  |
| Weekend warriors | 0.75 (0.47, 1.20) | 0.237 |  |
| Regularly active | 0.63 (0.53, 0.74) | <0.001 |  |
| **Active group defined as ≥200** **minutes/week of MVPA** | | | |
| Inactive | Reference |  |  |
| Insufficiently active | 0.74 (0.54, 1.02) | 0.062 |  |
| Weekend warriors | 0.74 (0.46, 1.18) | 0.208 |  |
| Regularly active | 0.63 (0.53, 0.74) | <0.001 |  |

All analyses were adjusted for age, gender, marital status, educational attainment, place of residence, employment status, smoking status, drinking status, night sleep duration, body mass index (BMI), and number of comorbidities. MVPA moderate-to-vigorous physical activity, OR odd ratio, CI confidence interval.

**Table S4. Associations between MVPA patterns and possible/confirmed sarcopenia among older adults.**

|  | **MVPA patterns** | **Model 1**  **OR (95% CI)** | ***P-*values** | **Model 2**  **OR (95% CI)** | ***P-*values** | **Model 3**  **OR (95% CI)** | ***P-*values** |
| --- | --- | --- | --- | --- | --- | --- | --- |
| **Possible sarcopenia** | Inactive | Reference |  | Reference |  | Reference |  |
|  | Insufficiently active | 0.73 (0.52, 1.03) | 0.069 | 0.83 (0.58, 1.18) | 0.292 | 0.77 (0.54, 1.11) | 0.166 |
|  | Weekend warriors | 0.42 (0.28, 0.64) | <0.001 | 0.56 (0.37, 0.86) | 0.009 | 0.58 (0.37, 0.89) | 0.014 |
|  | Regularly active | 0.47 (0.40, 0.54) | <0.001 | 0.61 (0.51, 0.72) | <0.001 | 0.63 (0.53, 0.75) | <0.001 |
|  |  |  |  |  |  |  |  |
| **Confirmed sarcopenia** | Inactive | Reference |  | Reference |  | Reference |  |
|  | Insufficiently active | 0.77 (0.44, 1.36) | 0.371 | 1.10 (0.60, 2.03) | 0.753 | 1.03 (0.52, 2.04) | 0.928 |
|  | Weekend warriors | 0.51 (0.25, 1.03) | 0.061 | 0.79 (0.38, 1.68) | 0.545 | 0.99 (0.44, 2.22) | 0.985 |
|  | Regularly active | 0.44 (0.34, 0.57) | <0.001 | 0.68 (0.50, 0.92) | 0.013 | 0.63 (0.45, 0.90) | 0.010 |

MVPA, moderate-to-vigorous physical activity; OR, odd ratio; CI, confidence interval.

Model 1: No covariates were adjusted.

Model 2: Adjusted for age, gender, marital status, educational attainment, place of residence, and employment status.

Model 3: Adjusted for Model 2 + smoking status, drinking status, night sleep duration, body mass index (BMI), and number of comorbidities.
